# American Medical Association

**Published:** 1890-05

**Authors:** William B. Atkinson

**Affiliations:** Permanent Secretary; Philadelphia, 1400 Pine Street, S. W. cor. Broad


### AMERICAN MEDICAL ASSOCIATION.

THE Forty-first Annual Session will be held in Nashville, Tenn., on Tuesday, Wednesday, Thursday, and Friday, May 20th, 21st, 22d and 23d, commencing on Tuesday at 11 A. M., under the Presidency of Dr. E. M. Moore, of Rochester, N. Y.

ADDRESSES.

On General Medicine, by Dr. N. S. Davis, Chicago, Ill.

On General Surgery, by Dr. Samuel Logan, New Orleans, La.

On State Medicine, by Dr. Alfred L. Carroll, New York, N. Y.

Committee on Arrangements: Dr. Wm. T. Briggs, Chairman, Nashville, Tenn.

SECTIONS.

"The Chairman of each Section shall prepare an address on the recent advancements in the branches belonging to his Section, including suggestions in regard to improvements in methods of work, and present the same to the Section over which he presides on the first day of its annual meeting. The reading of such address not to occupy more than forty minutes."—*By-Laws.*

Practice of Medicine, Materia Medica, and Physiology; Dr. J. H. Musser, Chairman, Philadelphia, Pa.,; Dr. H. McColl, Secretary, Lapeer, Mich.

Obstetrics and diseases of Women: Dr. W. W. Potter, Chairman, Buffalo, N. Y.; Dr. J. Hoffman, Secretary, Philadelphia, Pa.

Surgery and Anatomy: Dr. B. A. Watson, Chairman, Jersey City, N. J.; Dr. J. B. Deaver, Secretary, Philadelphia, Pa.

State Medicine: Dr. J. B. Hamilton, Chairman, Washington, D. C.; Dr. F. S. Bascum, Secretary, Salt Lake City, Utah.

Ophthalmology: Dr. S. C. Ayers, Chairman, Cincinnati, Ohio; Dr. E. J. Gardner, Secretary, Chicago, Ill.

Laryngology and Otology: Dr. J. O. Roe, Chairman, Rochester, N. Y.; Dr. F. H. Potter, Secretary, Buffalo, N. Y.

Diseases of Children: Dr. Isaac N. Love, Chairman, St. Louis, Mo.; Dr. E. F. Bush, Secretary, Mt. Vernon, N. Y.

Oral and Dental Surgery: Dr. J. L. Williams, Chairman, Boston, Mass.; Dr. E. S. Talbot, Secretary, Chicago, Ill.

Medical Jurisprudence: Dr. T. B. Evans, Chairman, Baltimore, Md.; Dr. T. D. Crothers, Secretary, Hartford, Ct.

Dermatology and Syphilography: Dr. ——, Chairman,——; Dr. W. T. Corlett, Secretary, Cleveland, Ohio.

"A member desiring to read a paper before a Section should forward the paper, or its *title and length* (not to exceed twenty minutes in reading), to the Chairman of the appropriate Section at least one month before the meeting."—*By-Laws.*

WILLIAM B. ATKINSON,
*Permanent Secretary.*

Philadelphia, 1400 Pine street, S. W. cor. Broad.

---

